# Supplementary material for: Alignment and mapping methodology influence transcript abundance estimation
Source: Genome Biol. 2020 Sep 7;21:239. doi: 10.1186/s13059-020-02151-8 (PMC7487471; doi:10.1186/s13059-020-02151-8)
Supplement: Supplementary file 1 — Additional file 1 Supplementary Material for “Alignment and mapping methodology influence transcript abundance estimation”. The file contains supplementary tables and figures for the manuscript. [file 13059_2020_2151_MOESM1_ESM.pdf]

Supplementary Material for “Alignment and mapping methodology influence transcript abundance estimation”

Avi Srivastava*[1], Laraib Malik*[1], Hirak Sarkar[2], Mohsen Zakeri[2], Fatemeh Almodaresi[2], Charlotte Soneson[3,4], Michael I. Love[5,6], Carl Kingsford[7], and Rob Patro[2, *]

[1] *Department of Computer Science, Stony Brook University*
[2] *Department of Computer Science, University of Maryland*
[3] *Friedrich Miescher Institute for Biomedical Research, Basel, Switzerland*
[4] *SIB Swiss Institute of Bioinformatics, Basel, Switzerland*
[5] *Department of Biostatistics, University of North Carolina at Chapel Hill*
[6] *Department of Genetics, University of North Carolina at Chapel Hill*
[7] *Computational Biology Department, School of Computer Science, Carnegie Mellon University*
[*] *Corresponding author: Rob Patro, rob@cs.umd.edu*

	Genome indexed	Alignment scoring	Indels allowed	CIGAR string	Quantification method
Bowtie2	✗	✓	✓	✓	Salmon
Bowtie2_strict	✗	✓	✗	✓	Salmon
Bowtie2_RSEM	✗	✓	✗	✓	RSEM
STAR	✓	✓	✓	✓	Salmon
STAR_strict	✓	✓	✗	✓	Salmon
STAR_RSEM	✓	✓	✗	✓	RSEM
quasi	✗	✗	✓	✗	Salmon
SA	✗*	✓**	✓	✗	Salmon
SAF	✓	✓**	✓	✗	Salmon

Table S1: Various factors altered under each pipeline. *Here, under SA, only regions of the genome that are sequence similar to the transcriptome are indexed, but not the whole genome. Refer to Section 4 for further details on how the sequences are obtained. **While SA and SAF produce alignment scores, they do not perform backtracing or reconstruct the edit operations that were used to obtain the optimal alignment score.

*Contributed equally.

Method	Truth
Bowtie2	0.909 ± 0.001
Bowtie2_strict	0.911 ± 0.001
Bowtie2_RSEM	0.917 ± 0.001
SA	0.914 ± 0.001
SAF	0.913 ± 0.001
quasi	0.888 ± 0.001
STAR	0.858 ± 0.001
STAR_strict	0.862 ± 0.001
STAR_RSEM	0.869 ± 0.002

Table S2: Spearman correlation against ground truth for data simulated using RSEM simulator.

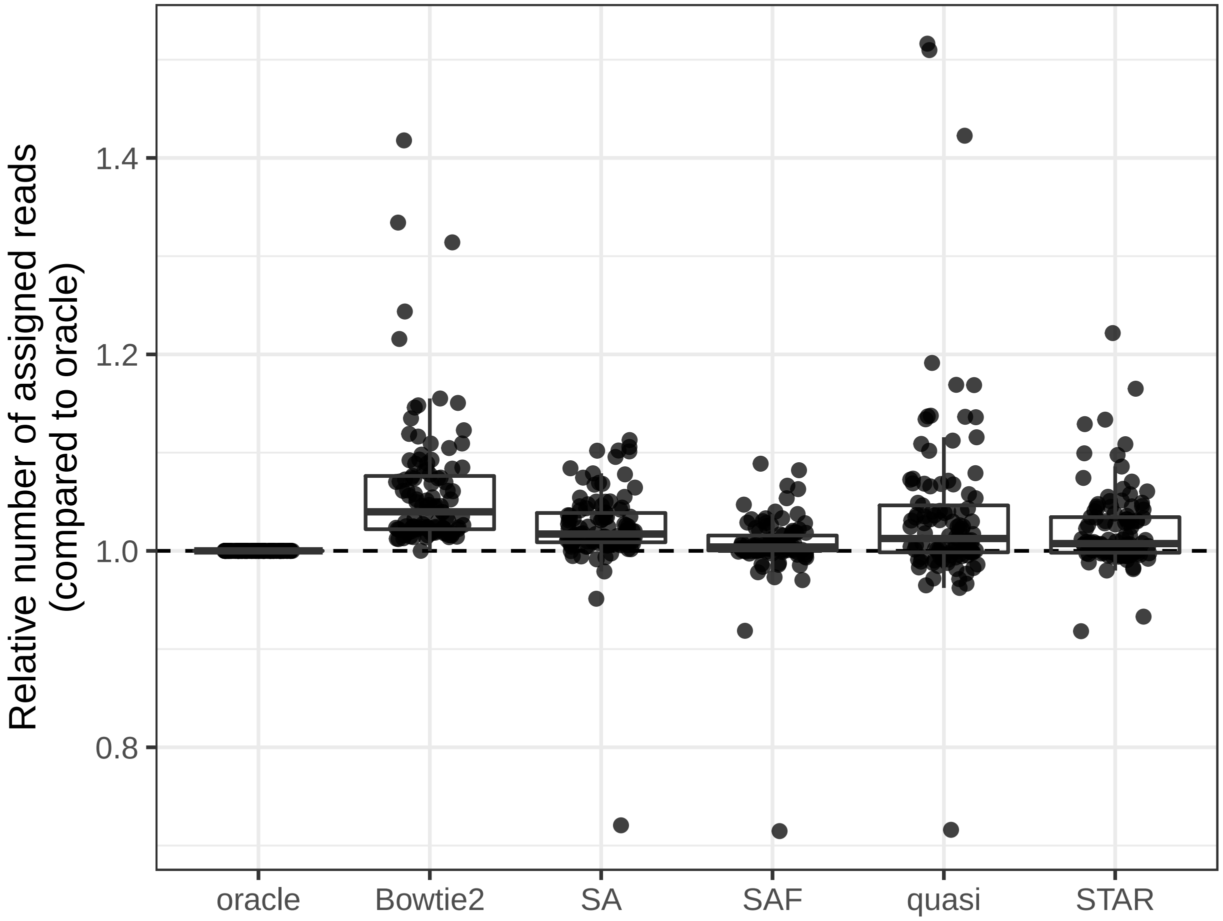

Fig. S1. Mapping rates of different methods, relative to oracle, for 109 experimental samples.

	Oracle	Bowtie2	SAF	SA	quasi	STAR
Oracle	1.000/0.000	0.838/0.051	0.900/0.031	0.850/0.045	0.799/0.060	0.856/0.040
Bowtie2	–	1.000/0.000	0.797/0.060	0.825/0.049	0.710/0.090	0.746/0.064
SAF	–	–	1.000/0.000	0.919/0.045	0.837/0.045	0.883/0.022
SA	–	–	–	1.000/0.000	0.789/0.075	0.807/0.052
quasi	–	–	–	–	1.000/0.000	0.824/0.047
STAR	–	–	–	–	–	1.000/0.000

Table S3: Mean/standard deviation of Spearman correlation between all methods on 40 single-cell experimental datasets after removing short transcripts with length < 300.

	Oracle	Bowtie2	SAF	SA	quasi	STAR
Oracle	1.000/0.000	0.926/0.037	0.956/0.021	0.934/0.021	0.846/0.061	0.939/0.027
Bowtie2	–	1.000/0.000	0.910/0.042	0.943/0.026	0.831/0.070	0.874/0.040
SAF	–	–	1.000/0.000	0.963/0.022	0.858/0.058	0.929/0.017
SA	–	–	–	1.000/0.000	0.859/0.057	0.897/0.024
quasi	–	–	–	–	1.000/0.000	0.836/0.057
STAR	–	–	–	–	–	1.000/0.000

Table S4: Mean/standard deviation of Spearman correlation between all methods on 69 bulk experimental datasets after removing short transcripts with length < 300.

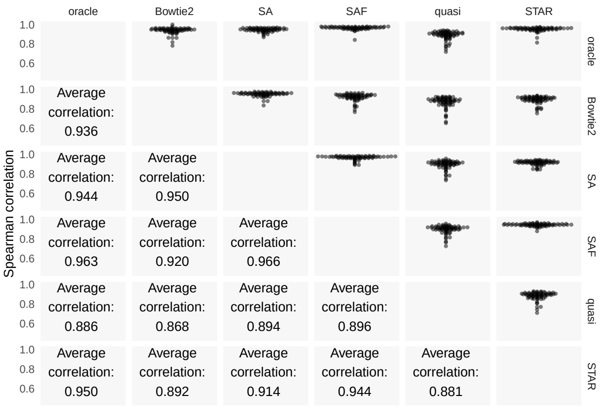

(a) Bulk

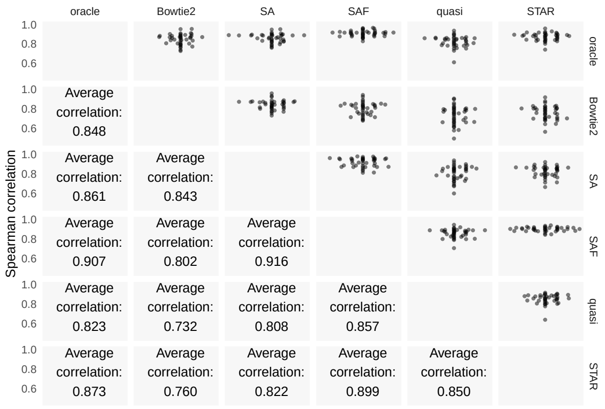

(b) Single-cell

Fig. S2. The upper triangle of the matrix shows swarm plots of pairwise correlations of read counts predicted by the different approaches on the experimental samples. The bottom half shows the average Spearman correlations between methods across the 109 bulk and single-cell samples.

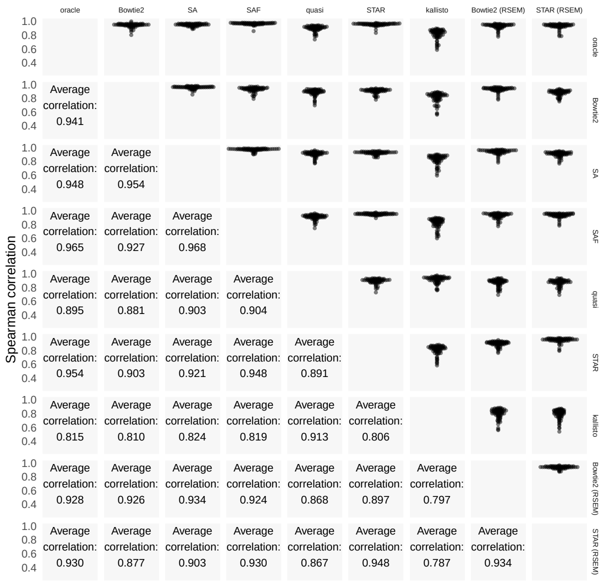

(a)

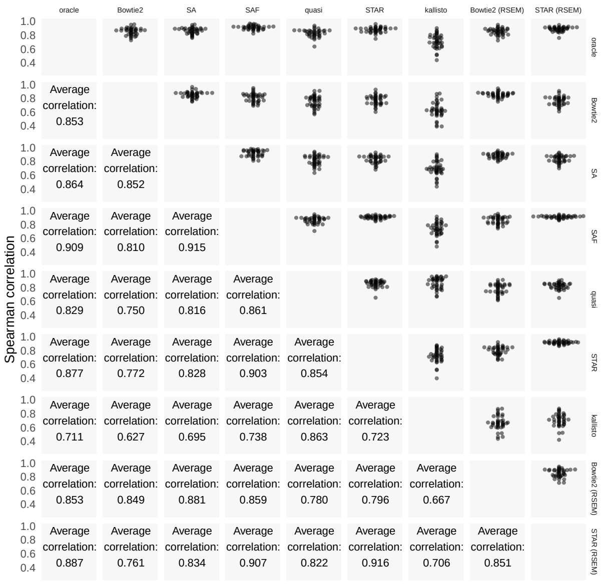

(b)

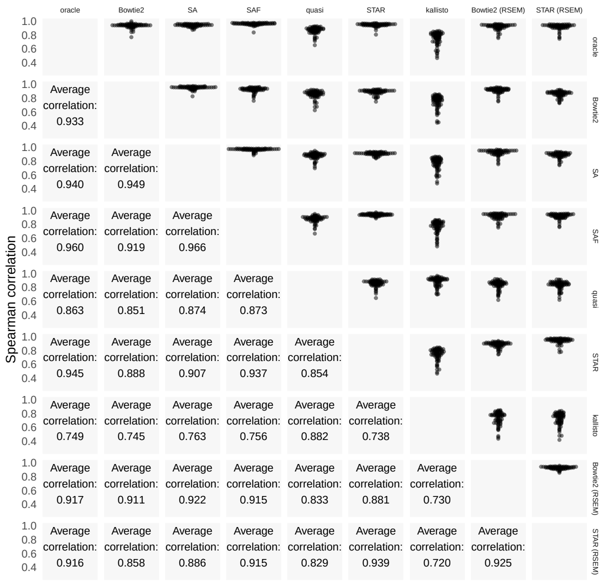

(c)

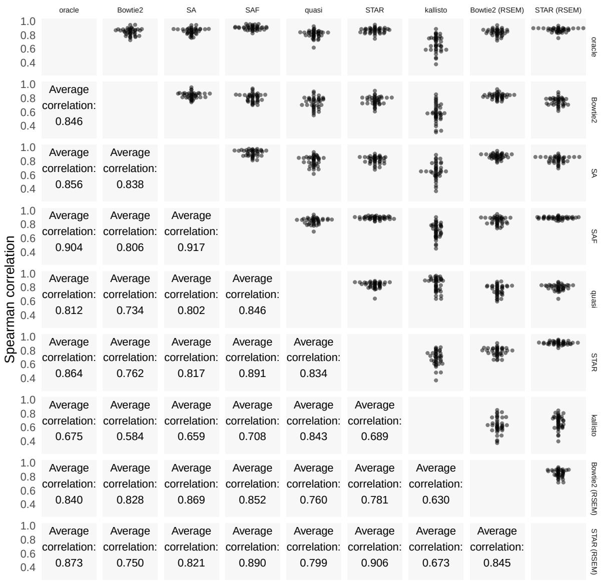

(d)

Fig. S3. The top half of each matrix shows swarm plots of the pairwise Spearman correlations using count (a) (b) and values for bulk and single-cell (respectively) (c) (d) TPM for bulk and single-cell (respectively) predicted by the different approaches on the experimental samples. The quantification method for each pipeline is the same, except kallisto and RSEM, where both the mapping and quantification algorithms are different. Hence, while other methods disallow orphaned reads and dovetailed mappings, the kallisto output will include them, which may explain, in part, the increased divergence from the alignment-based methods. Similarly, the RSEM based methods disallow indels in the read alignments.

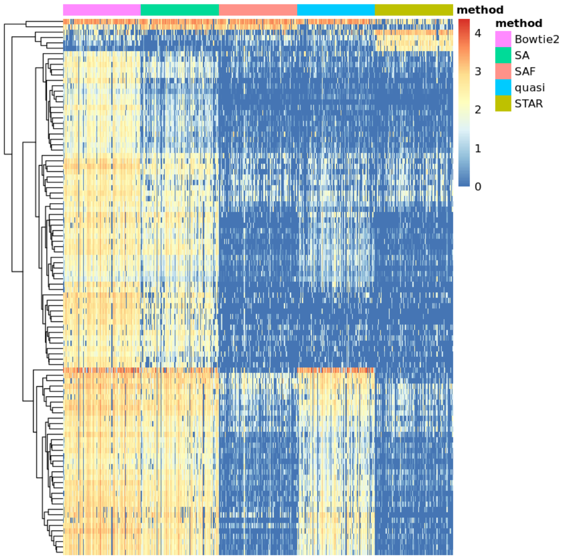

(a)

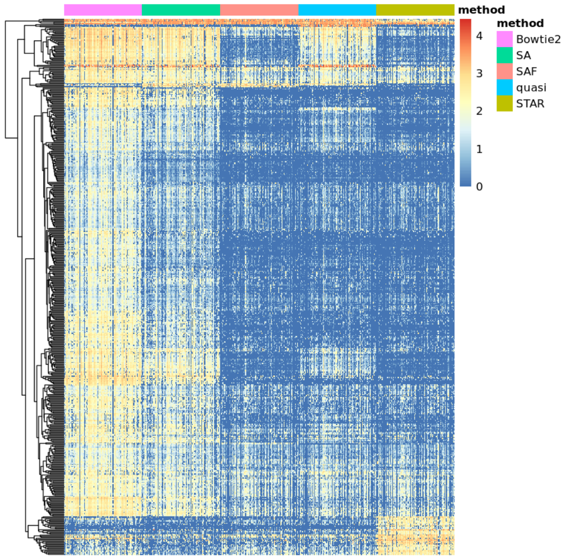

(b)

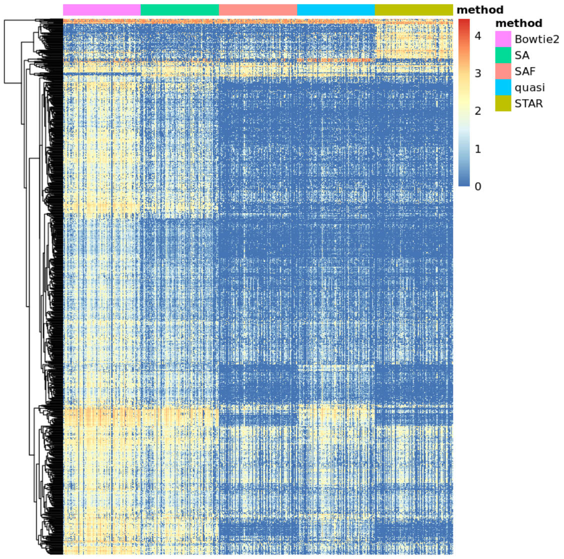

(c)

Fig. S4. The $\log_2$(CPM) for 109 samples grouped by method for the top 100 (a), 500 (b), and 1000 (c) differential transcripts. Limma-trend was used with `scaledTPM` counts (generating counts from per-sample TPMs by scaling to the library size) via tximport [1], with a `prior.count` of 3, and using a design of ~`sample + method`. An F-statistic was generated by specifying coefficients representing differences among the methods and the top transcripts chosen using the F-test p-value.

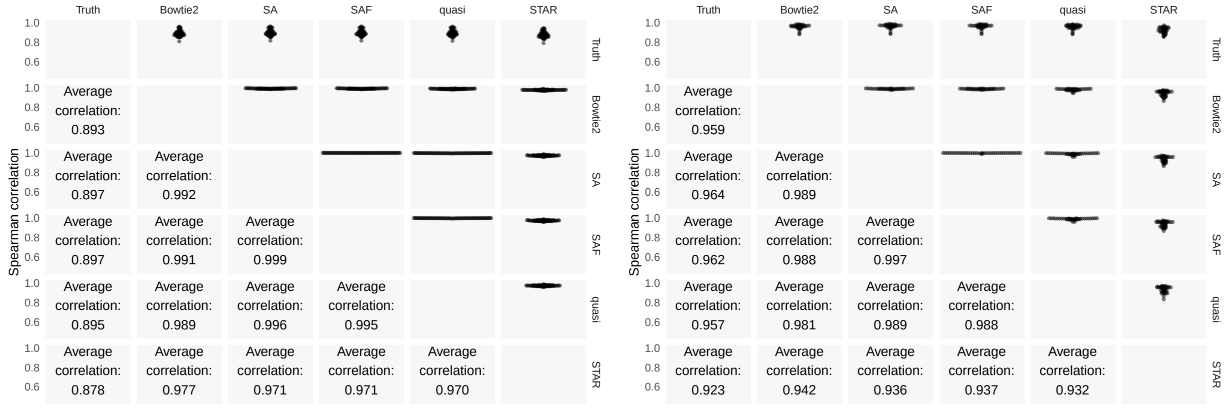

(a) Bulk

(b) Single-cell

Fig. S5. The top half of the matrix shows swarm plots of the pairwise correlations of TPM values predicted by the different approaches with each other and with the ground truth abundances on the simulated samples. The bottom half shows the average Spearman correlations between the different approaches across the 109 samples. The expected effective length of each transcript was computed according to the true fragment length distribution. Given the true fragment counts and expected effective lengths, the TPM is computed as in Li and Dewey [2].

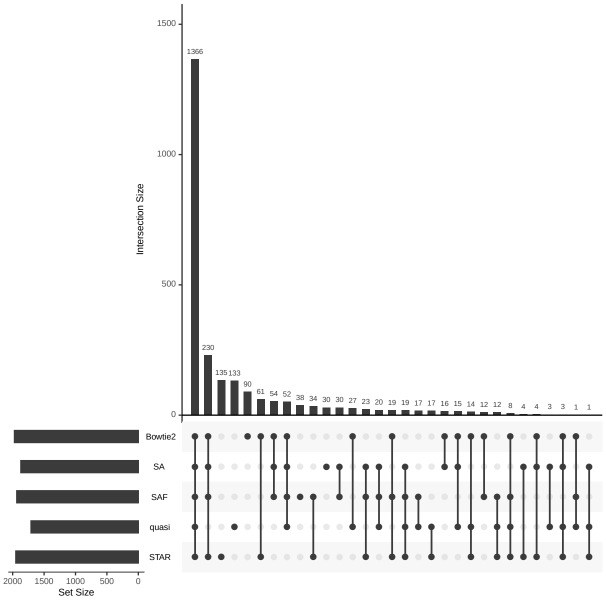

(a)

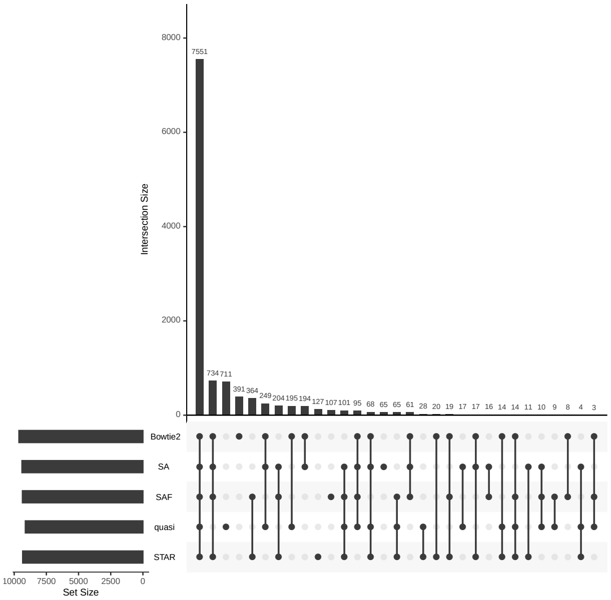

(b)

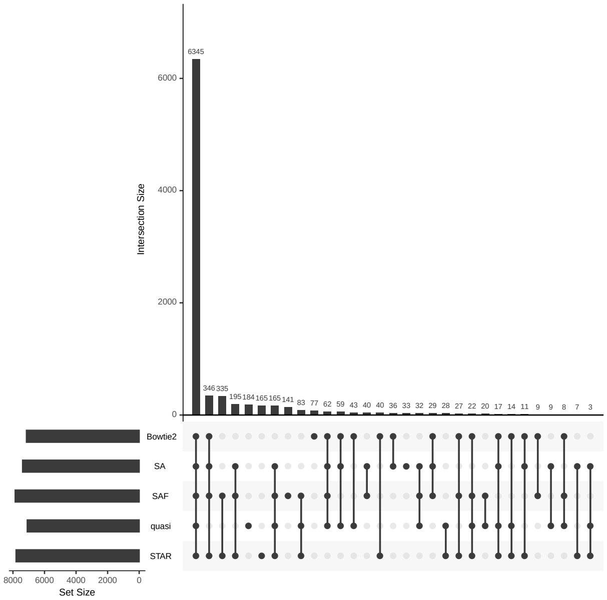

(c)

Fig. S6. Comparison of sets of differentially expressed genes, and their overlaps, computed using each method, when filtered at an FDR of 0.05.

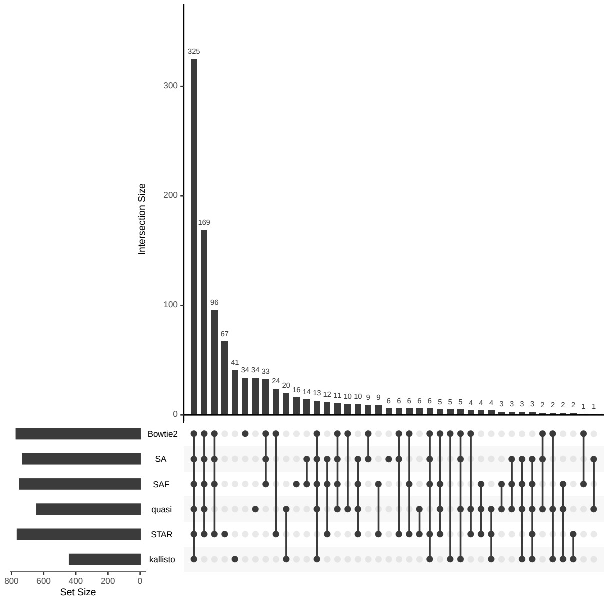

(a)

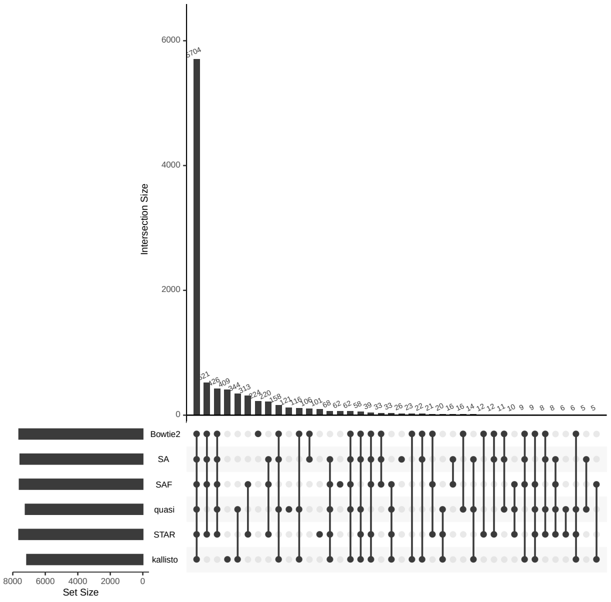

(b)

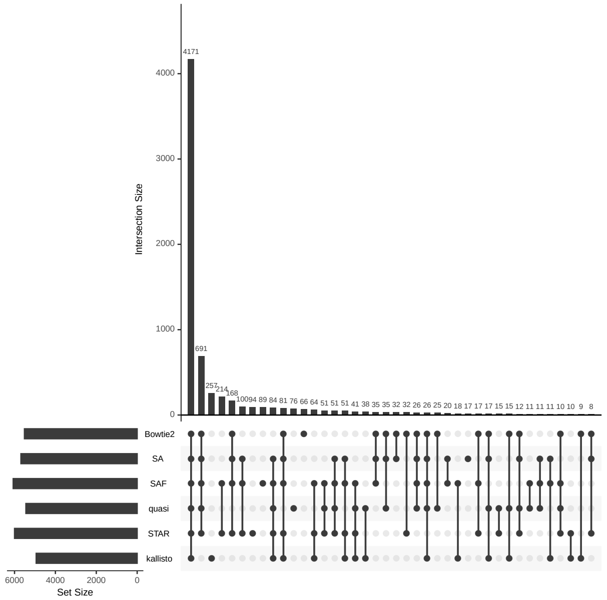

(c)

Fig. S7. Comparison of sets of differentially expressed genes, and their overlaps, computed using each method, at FDR 0.01 after including kallisto as an additional lightweight mapping approach.

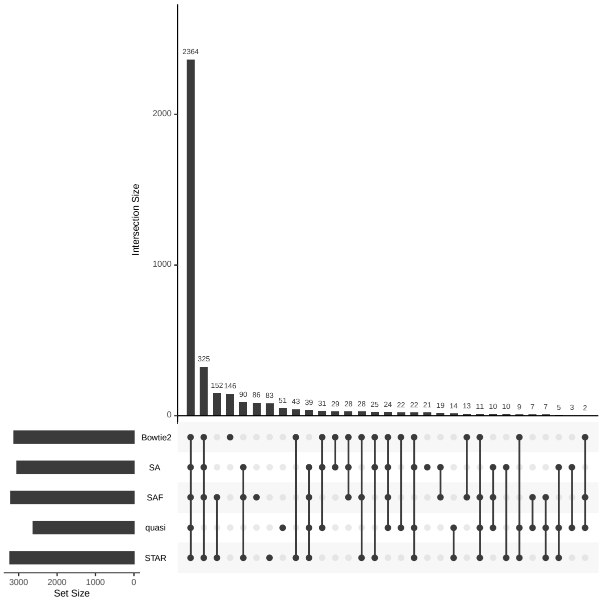

(a)

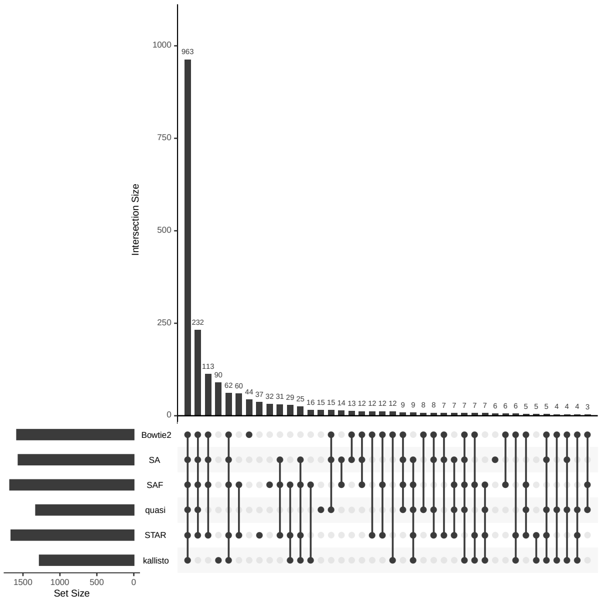

(b)

Fig. S8. Comparison of sets of differentially expressed transcripts, and their overlaps, computed using each method. Figure (a) shows the results when filtered at an FDR of 0.05 and (b) shows the results at FDR 0.01 after including kallisto as an additional lightweight mapping approach.

Bibliography

[1] Charlotte Soneson, Michael I Love, and Mark D Robinson. Differential analyses for RNA-seq: transcript-level estimates improve gene-level inferences [version 2; peer review: 2 approved]. *F1000Research*, 4:1521, 2016.

[2] Bo Li and Colin N Dewey. RSEM: accurate transcript quantification from RNA-Seq data with or without a reference genome. *BMC Bioinformatics*, 12(1):323, 2011.
