# Supplementary material for: Alignment and mapping methodology influence transcript abundance estimation
Source: Genome Biol. 2020 Sep 7;21:239. doi: 10.1186/s13059-020-02151-8 (PMC7487471; doi:10.1186/s13059-020-02151-8)
Supplement: Supplementary file 2 — Additional file 2 Review history. [file 13059_2020_2151_MOESM2_ESM.docx]

**Review History**

**First round of review**

**Reviewer 1**

The manuscript "Alignment and mapping methodology influence transcript abundance estimation" by Srivastava et al. describes the motivation for an enhanced version of their salmon software for abundance estimation, which leverages improved read alignments and decoy targets to improve on quantification.  Both simulated and real rna-seq data are leveraged for benchmarking quantification accuracy with comparison to alternative methods.

Salmon is a highly popular software for rna-seq based transcript quantification and these software enhancements involving 'selective alignment' (SA[F]) are welcomed additions. While I appreciate the efforts in the manuscript to motivate their need and demonstrate impact, I find certain shortcomings that could be addressed.

The accuracy comparisons are performed using simulated and real rna-seq data, and both are problematic in various ways, some of which the authors address in the manuscript.  The authors note that the simulated data is not a good reflection of real rna-seq data and that this is due to current simulation software not accounting for many of the greater complexities of real rna-seq data. Hence, the takeaways from benchmarking using their simulated data have limited value.  The authors were able to delve into the effects of indels on quantification accuracy using the simulated data. While the analysis into the effects of indels in quantification accuracy was useful, the likely more relevant issue that was a major motivation behind salmon SA[F] has to do with read mismapping, and this was addressed only anecdotally.  Improvements to the simulations should greatly benefit this section of the manuscript. For example, instead of simulating reads from only the reference transcripts, they could first identify and reconstruct all transcriptionally active regions of the genome (using tools like stringtie, augmented with the reference isoforms), simulate reads from those reconstructed isoforms, and then quantify accuracy levels.  Such simulations would include reads derived from unspliced introns, pseudogenes, paralogs, etc., and yield simulations that should be more on par with real rna-seq data, but with the key benefit of knowing where each simulated read was derived.  The impacts of read mismappings and the value of salmon SA[F] should be readily apparent in the context of such simulated data.

For the experiments with real rna-seq data, there was no clear ground truth for benchmarking, and no experimental data (ie. qrt-pcr) for evaluating isoform expression quantification accuracy. Hence, the authors set about to operationally define an 'oracle' reference quantification for which all methods could be compared.  The oracle was defined by restricting trusted read alignments to the subset of bowtie2 alignments that mapped with higher alignment scores to reference transcripts than to the reference genome (as by the STAR aligner).  The oracle quantifications were performed using salmon.  The fact that salmon was being used to define the oracle reference quantification to which all other tools are then compared (including salmon in different execution modes) raises a red flag.  Ideally, some orthogonal trusted method would be used to define the oracle.  

The absence of kallisto as one of the comparators is a glaring omission.  Since kallisto, to my knowledge, lacks a similar mechanism to leverage decoy sequences as in salmon SA, but is otherwise similar to salmon in other respects, this would be an opportunity to further highlight salmon's extended capabilities.  Furthermore, it is unfortunate that the authors eliminated RSEM from the applications on real rna-seq data.  They justify excluding RSEM due to it requiring indel-free alignments and their earlier evidence that it is important to leverage indel-containing alignments for improved quantification.  However, RSEM continues to be widely used and is widely perceived as a critical standard in quantification method comparisons. If indel-containing alignments are significantly confounding in quantification of real data, then this should be shown here as well.

For the experiments performed using real rna-seq data, it is entirely unclear why the authors chose to leverage single cell RNA-seq data for many of their samples, other than they may have just happened to appear within a list of random sets queried from SRA.  As the authors well know, quantification of single cell RNA-seq data has complexities and challenges that differ quantification of bulk data, largely stemming from differences in RNA quality and quantity in single cell transcriptomes.  Given that the authors do not have convincing evidence for establishing reference truth sets in single cell data, it would behoove the authors to restrict their study to bulk samples.  Otherwise, it would be justified for the authors to specifically address the differences between bulk and single cell data and issues regarding PCR duplicates, drop-out, and single-cell specific quantification considerations for accuracy assessments.

The impact on differential expression analysis is provided to further demonstrate the effect of quantification methods on downstream analyses.  Here, again since it's real data, there is no clear ground truth, and so while it can be shown how the different quantifications impact findings, it's not possible to unambiguously characterize calls as false positives or false negatives - as you could with simulated data.  Since the main point of this section is that lightweight mapping tends to miss the largest number of genes, it would be particularly helpful to have another lightweight mapper besides salmon in quasi-alignment mode, more specifically, including kallisto to show that this is a feature of lightweight mappers and not restricted to salmon's light-weight mapping mode.

Minor:

The bulleted list in section 2.1 might be better served as a table with columns indicating the alignment mode, quantification mode, and other properties.

For simulating reads, instead of restricting yourself to polyester, perhaps try flux simulator, RSEM's simulator, or other, to further demonstrate that the findings are not coupled to a specific read simulator and instead are tied to the quantification method as indicated.

Table 1 and 2 have identical legend titles.

Sections 2.6 and 2.7 could be moved to supp. materials, if there's a need to shorten the paper.

**Reviewers 2**

Srivistava et al. present a nice comparison of mapping strategies using both simulated and real datasets to quantify the impact that alignment choices can have on downstream analyses of RNA-seq data such as abundance estimation and differential expression analysis. In addition, they present a new mapping strategy, selective alignment, that they benchmark against 3 popular strategies: Bowtie, STAR, and lightweight mappers.  In particular, they highlight the limitations of benchmarking based on simulated datasets alone, showing that some key features of experimental data are missing from simulated datasets, even when real data is used to seed the simulations. Most of their conclusions can be summed up in the following two sentences: 1) True alignment methods tend to do better than lightweight mappers. 2) Even true alignment methods can differ from each other in terms of the cases in which they report false positive and negative alignments.

I think this is an important paper for those in the field, especially as the scale of single-cell RNA-seq experiments have pushed many groups to use the faster lightweight mapping strategies.  However, I do have a couple of major and minor questions that I think should be addressed by the authors.

1) Many of the comments on the differences between simulations and real experimental data center on the difficulty of having a true validation set for experimental data, hence their reliance on the "oracle set" created from the consensus of methods on the experimental data. So, I'm wondering if the authors tried using the experimental dataset from the SEQC Consortium (MAQC-III, Nature Biotech 2014; PMC4321899). If they deem this dataset unhelpful for this application, could they explain why they chose not to present comparisons to this data?

2) I like the selective alignment strategy (SA) given that it particularly addresses the need to acknowledge alignments to unannotated or unusual genomic transcripts. I think it would be nice if the authors could put a bit more details of this methods and its impact on downstream work. It's unclear to me if their decoy strategy for the SA and SAF methods detects but then discards these alignments before continuing downstream, or if these are rather retained in alignment files, but simply not included in the estimates for annotated sequence-similar transcripts? I suspect these represent not just alternatively spliced introns, but also intronic reads from transcripts (lncRNAs and transposons) not in their GTF files. Additionally, I would expect pseudogenes, short stretches of STR regions, and expressed intergenic transposons to be a large source of the sequence-similar transcripts that STAR finds but not Bowtie. Could the authors discuss how these are treated and whether unexpected reads from these regions are rescuable from the SA strategies for analysis using expanded annotation files?

3) Given the utility of fast, accurate lightweight methods for single-cell studies, I think the authors should spend a little time in the discussion talking about this being an important application where their SA methods would be particularly useful. It's hinted at in Fig 3B, but then not discussed much elsewhere.

4) Minor: I'm a little perplexed at the switch up on methods/data for Figures 5 & 6. They use DESeq for gene-level analysis on 2 datasets in Fig 5, then switch to Sleuth for transcript-level analysis in Fig 6 on a third dataset. I think it would be nicer to see a comparison of gene-level and transcript-level analyses on the same data.  E.g., do you see the same results for relative method performance regardless of whether you're looking at gene-level or transcript-level results? Or, does this move around more?  Since they don't have validation at either gene or transcript level anyway, and aren't comparing to the conclusions drawn from the original papers for these studies, it seems strange to keep moving the target.

**Authors’ response to reviewers**

Reviewer 1

Reviewer reports:

Reviewer #1: The manuscript "Alignment and mapping methodology influence transcript abundance estimation" by Srivastava et al. describes the motivation for an enhanced version of their salmon software for abundance estimation, which leverages improved read alignments and decoy targets to improve on quantification. Both simulated and real rna-seq data are leveraged for benchmarking quantification accuracy with comparison to alternative methods.

* Salmon is a highly popular software for rna-seq based transcript quantification and these software enhancements involving 'selective alignment' (SA[F]) are welcomed additions. While I appreciate the efforts in the manuscript to motivate their need and demonstrate impact, I find certain shortcomings that could be addressed.

==> We thank the reviewer for the detailed and insightful comments and feedback on the manuscript. We have attempted to address all of the reviewers’ concerns, as detailed below.

* The accuracy comparisons are performed using simulated and real rna-seq data, and both are problematic in various ways, some of which the authors address in the manuscript. The authors note that the simulated data is not a good reflection of real rna-seq data and that this is due to current simulation software not accounting for many of the greater complexities of real rna-seq data. Hence, the takeaways from benchmarking using their simulated data have limited value. The authors were able to delve into the effects of indels on quantification accuracy using the simulated data. While the analysis into the effects of indels in quantification accuracy was useful, the likely more relevant issue that was a major motivation behind salmon SA[F] has to do with read mismapping, and this was addressed only anecdotally. Improvements to the simulations should greatly benefit this section of the manuscript. For example, instead of simulating reads from only the reference transcripts, they could first identify and reconstruct all transcriptionally active regions of the genome (using tools like stringtie, augmented with the reference isoforms), simulate reads from those reconstructed isoforms, and then quantify accuracy levels. Such simulations would include reads derived from unspliced introns, pseudogenes, paralogs, etc., and yield simulations that should be more on par with real rna-seq data, but with the key benefit of knowing where each simulated read was derived. The impacts of read mismappings and the value of salmon SA[F] should be readily apparent in the context of such simulated data.

==> We have performed tests using the suggested method, with simulated data based on StringTie2-assembled transcripts. The details and results from this are included in Section 2.3 of the manuscript. These new results highlight, as expected, that unannotated transcription as captured by StringTie2 has a negative effect on the accuracy of transcript-level quantification. The simulation results also buttress the results on experimental data by demonstrating, in the presence of a ground truth, that the SAF methodology attains the greatest accuracy (followed by alignment to the genome using STAR).
We would like to thank the reviewer for this insightful suggestion. However, we would also like to emphasize that while we highlight the shortcomings of existing simulation methods in the manuscript, improving the realism of simulations generally is an extensive endeavor that requires more research and is well beyond the scope of this paper. Please note that we also changed the SAF results in Section 2.3 after correcting a mistake in our pipeline. This change does not affect any of the previous observations and conclusions.

* For the experiments with real rna-seq data, there was no clear ground truth for benchmarking, and no experimental data (ie. qrt-pcr) for evaluating isoform expression quantification accuracy. Hence, the authors set about to operationally define an 'oracle' reference quantification for which all methods could be compared. The oracle was defined by restricting trusted read alignments to the subset of bowtie2 alignments that mapped with higher alignment scores to reference transcripts than to the reference genome (as by the STAR aligner). The oracle quantifications were performed using salmon. The fact that salmon was being used to define the oracle reference quantification to which all other tools are then compared (including salmon in different execution modes) raises a red flag. Ideally, some orthogonal trusted method would be used to define the oracle.

==> With regards to the reviewer’s concern about using experimental data for benchmarking, we would like to share that while performing this study we searched for various datasets where RNA-seq was paired with experiments such as qrt-PCR. However, such experiments generally focus on a small number of genes (typically, not transcripts) that have very specific, well-annotated sequences. Hence, multi-mapping reads and spurious mappings are much less likely to affect such targets. As a result, most RNA-seq pipelines perform similarly on these genes. This is how, after considerable effort in exploring alternative approaches, we came to the idea of constructing an oracle.
The oracle is based on quantification estimates from salmon because this study is focused strictly on the effect of alignment strategies on quantification. Hence, we have kept the quantification method consistent throughout the project. We are not validating the quantification method. That is, the main point of the manuscript is not to compare salmon against other quantification tools, but rather to explore the effect of alignment and mapping methodology on quantification performance while holding the specific quantification methodology fixed. Also, as we describe in Section 2.1 of the manuscript, to the best of our knowledge, no other quantification method besides salmon can process both alignment and lightweight-mapping outputs. Again, the purpose of this study is not to show that one quantification method is better than another but, instead, to show that alignment and mapping strategies have a significant effect on quantification, irrespective of the quantification method. This is also why quantification methods other than salmon have been placed in the Supplementary Material, to convey a clearer picture of the purpose of the study.

* The absence of kallisto as one of the comparators is a glaring omission. Since kallisto, to my knowledge, lacks a similar mechanism to leverage decoy sequences as in salmon SA, but is otherwise similar to salmon in other respects, this would be an opportunity to further highlight salmon's extended capabilities. Furthermore, it is unfortunate that the authors eliminated RSEM from the applications on real rna-seq data. They justify excluding RSEM due to it requiring indel-free alignments and their earlier evidence that it is important to leverage indel-containing alignments for improved quantification. However, RSEM continues to be widely used and is widely perceived as a critical standard in quantification method comparisons. If indel-containing alignments are significantly confounding in quantification of real data, then this should be shown here as well.

==> We would like to point out to the reviewer that kallisto has not been omitted from the analysis. All results with kallisto are present in the Supplementary Material (and were present in the initial submission as well). The reason we have placed these results in the Supplementary Material is because these comparisons alter the quantification methodology itself, in addition to the alignment or mapping methodology. In the revised manuscript, at the reviewer’s request, we have also added RSEM quantification to Supplementary Material for Section 2.4. Despite the caveats that we place on comparisons that alter both the alignment methodology and the quantification methodology, we agree with the reviewer that the popularity of these other approaches likely makes it worthwhile to include a comparison, in the Supplementary Material, for interested readers. We have also tried to highlight this reasoning more in the manuscript so that it is clearer for the reader as well.

* For the experiments performed using real rna-seq data, it is entirely unclear why the authors chose to leverage single cell RNA-seq data for many of their samples, other than they may have just happened to appear within a list of random sets queried from SRA. As the authors well know, quantification of single cell RNA-seq data has complexities and challenges that differ quantification of bulk data, largely stemming from differences in RNA quality and quantity in single cell transcriptomes. Given that the authors do not have convincing evidence for establishing reference truth sets in single cell data, it would behoove the authors to restrict their study to bulk samples. Otherwise, it would be justified for the authors to specifically address the differences between bulk and single cell data and issues regarding PCR duplicates, drop-out, and single-cell specific quantification considerations for accuracy assessments.

==> We would like to clarify that the single-cell studies included under real data refer to full-length single-cell data (e.g. Smart-seq2 and related protocols) which is frequently aligned and quantified using bulk RNA-seq methods like those we have included here. We apologize for not clarifying this fully in the previous version of the manuscript, and have added text to do so, with citations of studies that have previously used these methods in the context of full-length single-cell RNA-seq. Finally, we believe it is important to include these single-cell results in the manuscript, and to analyze them along with, but separately from, the bulk RNA-seq results. Due to the limited read depth in full-length single-cell protocols, it is conceivable that read alignment accuracy is even more crucial in these data than in bulk RNA-seq (as there are fewer other reads to allow methods to statistically “correct” for spurious or incomplete alignment). Since these approaches are being used in full-length single-cell RNA-seq studies, we feel it is important to include such an analysis in the manuscript.


* The impact on differential expression analysis is provided to further demonstrate the effect of quantification methods on downstream analyses. Here, again since it's real data, there is no clear ground truth, and so while it can be shown how the different quantifications impact findings, it's not possible to unambiguously characterize calls as false positives or false negatives - as you could with simulated data. Since the main point of this section is that lightweight mapping tends to miss the largest number of genes, it would be particularly helpful to have another lightweight mapper besides salmon in quasi-alignment mode, more specifically, including kallisto to show that this is a feature of lightweight mappers and not restricted to salmon's light-weight mapping mode.

==> The kallisto results for differential expression are included in the Supplementary Material as well, for the same reasons described above. We also have added text in Section 2.4 of the main paper to show that the conclusion (in terms of performance of lightweight mapping methods) still holds after including kallisto.

Minor:

* The bulleted list in section 2.1 might be better served as a table with columns indicating the alignment mode, quantification mode, and other properties.

==> Thank the reviewer for this suggestion. Table S1 in the Supplementary Material presents this information in tabular form.

* For simulating reads, instead of restricting yourself to polyester, perhaps try flux simulator, RSEM's simulator, or other, to further demonstrate that the findings are not coupled to a specific read simulator and instead are tied to the quantification method as indicated.

==> We have included results for the first experiment using the RSEM simulator in Supplementary Material for Section 2.2, and observe broadly similar trends.

* Table 1 and 2 have identical legend titles.

==> We apologize for this error and have fixed the legend titles.

* Sections 2.6 and 2.7 could be moved to supp. materials, if there's a need to shorten the paper.

==> We believe there is not a page limit and hence have kept the structure of the manuscript. Of course, we will move content to the Supplementary Material as suggested by the reviewers and the editor if deemed necessary.


Reviewer 2


* Srivistava et al. present a nice comparison of mapping strategies using both simulated and real datasets to quantify the impact that alignment choices can have on downstream analyses of RNA-seq data such as abundance estimation and differential expression analysis. In addition, they present a new mapping strategy, selective alignment, that they benchmark against 3 popular strategies: Bowtie, STAR, and lightweight mappers. In particular, they highlight the limitations of benchmarking based on simulated datasets alone, showing that some key features of experimental data are missing from simulated datasets, even when real data is used to seed the simulations. Most of their conclusions can be summed up in the following two sentences: 1) True alignment methods tend to do better than lightweight mappers. 2) Even true alignment methods can differ from each other in terms of the cases in which they report false positive and negative alignments.

==> We thank the reviewer for the useful feedback and remarks on the work. We have attempted to address all of the reviewers’ concerns as detailed below.

* I think this is an important paper for those in the field, especially as the scale of single-cell RNA-seq experiments have pushed many groups to use the faster lightweight mapping strategies. However, I do have a couple of major and minor questions that I think should be addressed by the authors.

1) Many of the comments on the differences between simulations and real experimental data center on the difficulty of having a true validation set for experimental data, hence their reliance on the "oracle set" created from the consensus of methods on the experimental data. So, I'm wondering if the authors tried using the experimental dataset from the SEQC Consortium (MAQC-III, Nature Biotech 2014; PMC4321899). If they deem this dataset unhelpful for this application, could they explain why they chose not to present comparisons to this data?

==> We considered using SEQC samples, however we decided that these samples were not the ideal choice for understanding how alignment of reads from a diploid transcriptome affects quantification, as they are not samples from an organism with a diploid transcriptome. In SEQC, the "A" sample corresponds to universal human reference RNA (UHRR) and the "B" sample corresponds to human brain tissue reference RNA (HBRR) (while the C and D samples represent fixed mixes of A and B). UHRR is a mixture of total RNA from 10 cell lines of different origin, all of which are cancer cell lines and have substantial genomic rearrangements and somatic mutations compared to a non-tumor genome. Less information is available about HBRR, but it is also described by vendors such as ThermoFisher as reference RNA from pooled samples, so comprising RNA from brain tissue of some number of individuals with different genomes. As the focus of the paper is on alignment of RNA-seq reads for non-tumor samples representing individual organisms, we restricted our evaluation to samples of this type. We further did not consider the C or D samples of SEQC to assess quantification accuracy, as systematic bias in quantification of A and B samples may lead to internally consistent, but nevertheless inaccurate, quantification of C and D.

* 2) I like the selective alignment strategy (SA) given that it particularly addresses the need to acknowledge alignments to unannotated or unusual genomic transcripts. I think it would be nice if the authors could put a bit more details of this methods and its impact on downstream work. It's unclear to me if their decoy strategy for the SA and SAF methods detects but then discards these alignments before continuing downstream, or if these are rather retained in alignment files, but simply not included in the estimates for annotated sequence-similar transcripts? I suspect these represent not just alternatively spliced introns, but also intronic reads from transcripts (lncRNAs and transposons) not in their GTF files. Additionally, I would expect pseudogenes, short stretches of STR regions, and expressed intergenic transposons to be a large source of the sequence-similar transcripts that STAR finds but not Bowtie. Could the authors discuss how these are treated and whether
unexpected reads from these regions are rescuable from the SA strategies for analysis using expanded annotation files?

==> We thank the reviewer for the excellent suggestion. In the initial implementation of SA and SAF, the focus was entirely on preventing these reads, that do not originate from annotated transcripts, from adversely affecting the estimation of their abundance. Hence, the behavior was simply to discard these reads from consideration (i.e. don’t count them when performing transcript abundance inference), and only to keep track of the number of such reads that are encountered (Salmon provides, in a file called aux_info.json, statistics about how many reads were not quantified because they were best mapped to decoy sequence) .

However, since the reads that are determined to arise from decoys undergo the entire selective-alignment procedure (i.e. they are subject to mapping and alignment scoring validation), and hence their alignment positions to the decoys and scores are known, there is no reason not to propagate this information downstream if requested by the user. Hence, we are implementing this functionality and now describe it briefly in the paper. The user can choose to include the decoy mappings in the output SAM file. These mappings are denoted by a special flag `XT:A:D` (to make them easy to filter), and can then be used for subsequent analysis. We do wish to note that, unlike STAR, when aligning to the genome, the SAF strategy still looks for contiguous mappings of the reads to the underlying genome, so that if one is analyzing spliced features, they may want to re-align this small subset of reads using a splice-aware aligner. Nonetheless, we believe the marking and outputting of the decoy-aligned reads could prove a useful feature for users, so we are implementing this in the develop branch of salmon, and will include this capability in a point release before this paper is published (if the editor chooses to accept it). We have added a discussion of the handling of decoy-aligned sequences to the end of Section 4.3.

* 3) Given the utility of fast, accurate lightweight methods for single-cell studies, I think the authors should spend a little time in the discussion talking about this being an important application where their SA methods would be particularly useful. It's hinted at in Fig 3B, but then not discussed much elsewhere.

==> We thank the reviewer for this insightful observation, and we strongly agree with this point. We have edited text to discuss a bit more about single-cell quantification using SAF. We note that we restrict our analysis in this paper to bulk and full-length single-cell protocols, since the quantification methods we adopt are used for this type of data but are not appropriate for tagged-end single-cell data. However, there is no reason to believe that similar issues related to mapping and alignment methodology would not also affect the processing of data from tagged-end single-cell protocols (though that analysis is outside the scope of the, already long, current manuscript).

* 4) Minor: I'm a little perplexed at the switch up on methods/data for Figures 5 & 6. They use DESeq for gene-level analysis on 2 datasets in Fig 5, then switch to Sleuth for transcript-level analysis in Fig 6 on a third dataset. I think it would be nicer to see a comparison of gene-level and transcript-level analyses on the same data. E.g., do you see the same results for relative method performance regardless of whether you're looking at gene-level or transcript-level results? Or, does this move around more? Since they don't have validation at either gene or transcript level anyway, and aren't comparing to the conclusions drawn from the original papers for these studies, it seems strange to keep moving the target.

==> We thank the reviewer for pointing out this inconsistency. For the datasets analyzed in Figure 5, we have focused on gene-level differential expression, as that is still, by far, the most common type of differential analysis. We included the zika dataset for Figure 6 as an example of transcript level differential testing, since this dataset has been previously analyzed at the transcript level, as well as to demonstrate that one may expect the types of differences we observe at the gene level to also persist at the transcript level (even when accounting for inferential uncertainty). In the revised manuscript, we have also included gene-level differential expression results for the zika dataset as well. We chose not to provide transcript-level differential analysis results for the other datasets both since those datasets were originally analyzed at the gene level, and also because they have low replicates per-condition (2 vs. 2 and 2 vs. 3), severely limiting the power for transcript-level analysis.

**Second round of review**

**Reviewer 1**

I appreciate the response to my earlier inquiries and I'm pleased to see that the authors have taken sincere efforts to address them in the revised manuscript.

I only have a few minor remaining comments:

page 6, ~ line 31: typo 'showg' -> 'shows'

page 6, ~ line 51: the term 'oracle' is mentioned here, long before the term is defined and so seems out of place.

page 9, ~ lines 49-52: the newly inserted text seems out of place in that paragraph.

**Reviewer 2**

The authors have addressed all of my comments. In particular, I appreciate the efforts of the authors to alter the software in order to address one of the points raised. I have no more concerns.
